# Exploring the influence of national music lessons on subjective well-being, self-esteem, and national identity among university students: a case study from China

**DOI:** 10.3389/fpsyg.2023.1151007

**Published:** 2023-06-19

**Authors:** Hongyu Fu, Jinmei Tu

**Affiliations:** ^1^School of Education, Guangzhou University, Guangzhou, China; ^2^School of Music and Dance, Guangzhou University, Guangzhou, China

**Keywords:** national music lessons, subjective well-being, national identity, self-esteem, university students

## Abstract

This study aims to explore the influence of national music lessons on university students’ subjective well-being, self-esteem, and national identity. A Chinese university provided four national music courses spanning 8 weeks. The students’ subjective well-being, self-esteem, and national identity were measured before the commencement of the courses (T1), the fourth week of the courses (T2), and post the completion of the courses (T3). A total of 362 participants completed the Positive and Negative Affect Scales, the Satisfaction with Life Scale, the Rosenberg Self-Esteem Scale, and the National Identity Scale at T1, T2, and T3. Results indicated that national music lessons could improve university students’ subjective well-being, yet there was no effect on their national identity or self-esteem. Although high national identity and high self-esteem predicted a greater level of subjective well-being, self-esteem and national identity did not affect the influence of national music lessons on subjective well-being. National music lessons were particularly beneficial to students with low and middle levels of subjective well-being, in comparison to those with higher levels of subjective well-being. This paper verifies an efficient method to bolster students’ subjective well-being that can be conducted in educational practices.

## 1. Introduction

### 1.1. Subjective well-being and music lessons

Happiness is a spiritual feeling that has been sought out by people all their lives, and it has a far-reaching impact on all areas of an individual’s life. The indicator of happiness in positive psychology is subjective well-being, which refers to people’s general evaluation of life quality according to their own standards and feelings (Diener, 1984). Diener (2000) has identified three aspects of subjective well-being: positive affect (PA), negative affect (NA), and satisfaction with life (SWL). A person’s subjective well-being is associated with better mental health and low levels of depression and anxiety (Burns et al., 2011). After conducting a study collecting students’ written reflections, Backman (2016) suggested that subjective well-being is connected to learning, school engagement, appreciation of subjects or lesson content, others’ happiness, and prosocial behavior in a bidirectional crossovers fashion. Therefore, enhancing students’ subjective well-being can bring about beneficial changes in many areas of their life.

Tejada-Gallardo et al. (2020) proposed the incorporation of effective interventions into the curriculum as a viable way to improve students’ mental health. Implementing music courses for college students may be a productive method to enhance their subjective well-being. On the one hand, music can be employed as a means to regulate emotions (Randall et al., 2014). Research from the past has indicated that music could have a beneficial impact on one’s mental health by improving their emotional experiences (Leung and Cheung, 2020). Music classes provide opportunities for students to unwind and alleviate stress by listening to music. As a prior investigation has demonstrated, attending school concerts could foster students’ subjective well-being (Kwon et al., 2020). On the other hand, music education has a positive effect on mental health, as reflected in the results of many previous studies (Osmanoglu and Yilmaz, 2019; Mehraban, 2020; Sun, 2022). Mehraban (2020) believed that incorporating music education programs as a distinct curriculum could be an approach to bolster the mental health of students. By offering music courses in colleges and universities, students can gain an appreciation of the aesthetic, humanistic, and inheritance values of music (Behr et al., 2016). National music courses may benefit university students’ subjective well-being as a form of the music curriculum.

### 1.2. National music lessons and national identity

National music is a genre of music exclusive to a national group, characterized by its national features (Wang, 2001). National music comes from the social life and productive labor of various ethnic groups with its unique musical style (Regev, 2007; Dong, 2015). For example, Chinese national music consists of folk songs, folk instrumental music, folk dance, opera music, and rap music (Du, 2006). Chinese national music lessons offer the chance to acquire knowledge of Chinese national music, admire its beauty, and participate in singing it. Instruction in Chinese national music can be accessed at various educational levels, such as primary, junior high, senior high, and college. Chinese national music is a long-standing symbol of Chinese culture (China Culture, 2021). Appreciating national music is not as boring as learning national culture and knowledge. As the carrier of national culture, national music is a more acceptable form of cultural communication.

Social identity theory suggests that group membership is a fundamental part of an individual’s identity and self-concept (Tajfel, 1982). National identity refers to one’s sense of mutual belonging and obligation to a national group, including the part of one’s cognition, emotion, and behavior that is due to membership in that national group (Newman, 2005; Ha and Jang, 2014). The development of ethnic identity is dependent upon two processes: National socialization, in which people gain the behaviors, perceptions, values, and attitudes of their national group, and enculturation, the process of familiarizing oneself with and accepting their traditional national culture (Phinney, 1989, 1990). In certain countries and regions, music education is actively contributing to the promotion of national identity and cultural development through national musical art (Ho and Law, 2020). Boer et al. (2013) considered that national music is a source of national identity construction. They have provided empirical evidence based on six cultures that national music preference is linked with one’s national identity. National music lessons offer students the chance to gain knowledge of national cultures, aiding them in developing a stronger sense of national identity. Thus, national music lessons may contribute to the national identity of university students in a positive manner.

### 1.3. Self-esteem and national music lessons

Self-esteem is an individual’s global judgment or overall evaluation of self-competence and self-value (Rosenberg, 1965). It has been demonstrated by a previous study that individuals with high self-esteem have better work performance, better interpersonal relationships, more happiness, and a healthier lifestyle (Baumeister et al., 2003). A meta-analysis has established that there is a correlation between self-esteem and one’s job satisfaction and performance (Judge and Bono, 2001). Self-esteem is not only closely related to one’s personal life but also to one’s national identity. Previous research has revealed that national socialization and national identity could bolster an individual’s self-esteem (Lee et al., 2018). The predictive effects of national socialization and identity on self-esteem have also been observed in samples from China (Kuang and Nishikawa, 2021). National music education has the potential to cultivate a sense of national identity, which may also boost students’ self-esteem. Several studies have indicated that music could be beneficial in improving a person’s self-esteem. Chen et al. (2016) conducted a study to assess the impact of group music therapy on the mental health of Chinese prisoners, focusing on anxiety, depression, and self-esteem. They determined that group music therapy is an effective approach to improving anxiety, depression, and self-esteem. Yücesan and Şendurur (2018) discovered that music therapy, poetry therapy, and creative drama had a beneficial effect on the self-esteem of university students. The investigation of Sun (2022) has uncovered that music education could strengthen college students’ self-efficacy and self-esteem, thus improving their mental health. Through the music education offered by a national music course, university students may experience an improvement in their self-esteem.

### 1.4. Current study

This study intends to examine the impact of national music lessons on national identity, self-esteem, and subjective well-being among university students. To accomplish the goal of this study, a longitudinal survey was conducted at a university in China to serve as a case study. This study evaluates the role of Chinese music lessons by analyzing the differences in national identity, self-esteem, and subjective well-being of college students at three-time points. Based on previously available evidence, the hypotheses can be drawn:

*H1*: National music lessons can enhance university students’ subjective well-being.

*H2*: National music lessons can cultivate university students’ national identity.

*H3*: National music lessons can improve university students’ self-esteem.

## 2. Materials and methods

### 2.1. Study design and participants

This study was conducted in September–November 2022 at a Chinese university. The university offered four Chinese national music optional courses for sophomores, juniors, and seniors. The course contents mainly included: (1) Learning the knowledge of folk songs, appreciating and singing folk songs; (2) appreciating and singing minority songs; (3) appreciating national dance music; (4) learning the knowledge of national special instrumental music, and admiring the compositions performed by national instrumental music. The courses spanned 8 weeks. Data collection occurred at three points: before the commencement of the courses (T1), the fourth week of the courses (T2), and post the completion of the courses (T3). Each data collection included the measurements of national identity, self-esteem, and subjective well-being.

G*Power 3.1.9.7 was employed to determine the minimum sample size necessary (Faul et al., 2007). In line with the study design, *a priori* power analysis of ANOVA (Repeated measures, within factors) was conducted, with an effect size of *f* = 0.15, α = 0.01 (i.e., significant level), and 1- β = 0.99 (i.e., statistical power). The outcome of the calculation revealed a sample size of 206. A total of 393 students who were enrolled in the Chinese national music lessons completed questionnaires at T1. Among them, 369 participants followed our survey at T2. After T3, we confirmed that only 362 participants had finished all three questionnaires. The age of the participants ranged from 18 to 24 (Mage = 20.06 ± 0.90). Regarding gender, there were 153 males (42.3%) and 209 females (57.7%). In terms of grades, 78 students (21.5%) were sophomores, 279 students (77.1%) were juniors, and 5 students (1.4%) were seniors. As for the major, 203 students (56.1%) were majoring in natural science and engineering, 143 students (39.5%) in humanities and social sciences, and 16 students (4.4%) in art and aesthetics. Furthermore, 91 students (25.1%) were only children, and 271 students (74.9%) had siblings.

### 2.2. Measures

#### 2.2.1. Positive and negative affect scale

The Positive and Negative Affect Scale (PANAS) was used to measure both positive and negative affect, which contains 10 items for positive affect and 10 items for negative affect (Watson et al., 1988). Huang et al. (2003) provided a Chinese version of PANAS. Participants were asked to rate each item on a 5-point Likert scale ranging from 1 (not at all) to 5 (extremely), to measure the extent to which they had experienced the affect in the past month. In this study, Cronbach’s alphas for the Positive Affect (PA) subscale were 0.88, 0.91, and 0.89 at T1, T2, and T3, respectively. For the Negative Affect (NA) subscale, Cronbach’s alphas were 0.89, 0.91, and 0.90 at T1, T2, and T3, respectively.

#### 2.2.2. Satisfaction with life scale

Satisfaction with life was assessed using the Satisfaction with Life Scale (SWLS; Diener et al., 1985). The Chinese version of SWLS was translated by Xiong and Xu (2009). The SWLS presented five statements to which participants responded with their level of agreement on a 7-point Likert scale, from 1 (strongly disagree) to 7 (strongly agree). In the present study, Cronbach’s alphas for the SWLS were 0.86, 0.88, and 0.87 at T1, T2, and T3, respectively.

#### 2.2.3. Rosenberg self-esteem scale

The Rosenberg Self-Esteem Scale (RSES) was implemented to measure self-esteem (Rosenberg, 1965). The Chinese translation of RSES has completed verification (Wang et al., 1999). RSES was composed of 10 items, each with a rating from 1 (strongly disagree) to 4 (strongly agree). Wu et al. (2017) ascertained that item 8 should be regarded as a positively worded item when evaluating Chinese samples. In this study, Cronbach’s alphas for the RSES were 0.90, 0.88, and 0.89 at T1, T2, and T3, respectively.

#### 2.2.4. National Identity Scale

National Identity was measured using the National Identity Scale (NIS; Dong et al., 2015). It consisted of three dimensions: exploration, affirmation, and confirmation. Exploration involves actively participating in activities to gain knowledge about the nation. Affirmation represents the degree of the individual’s acceptance of the group identity. Confirmation refers to the individual’s recognition and sense of mission for the nation. NIS consisted of fifteen items. Participants were asked to rate their agreement on a 4-point scale, ranging from 1 (strongly disagree) to 4 (strongly agree). In the current study, Cronbach’s alphas for the NIS were 0.89 at T1, and 0.91 at T2 and T3.

## 3. Results

Statistical analysis for this study was conducted using IBM SPSS, version 28.0. Referring to the method of the previous study (Haslam et al., 2009), SWB was calculated by standardizing the SWLS and the PANAS and then combining them (SWB = SWLS + PA − NA).

### 3.1. Subjective well-being of university students

**Figure 1:**
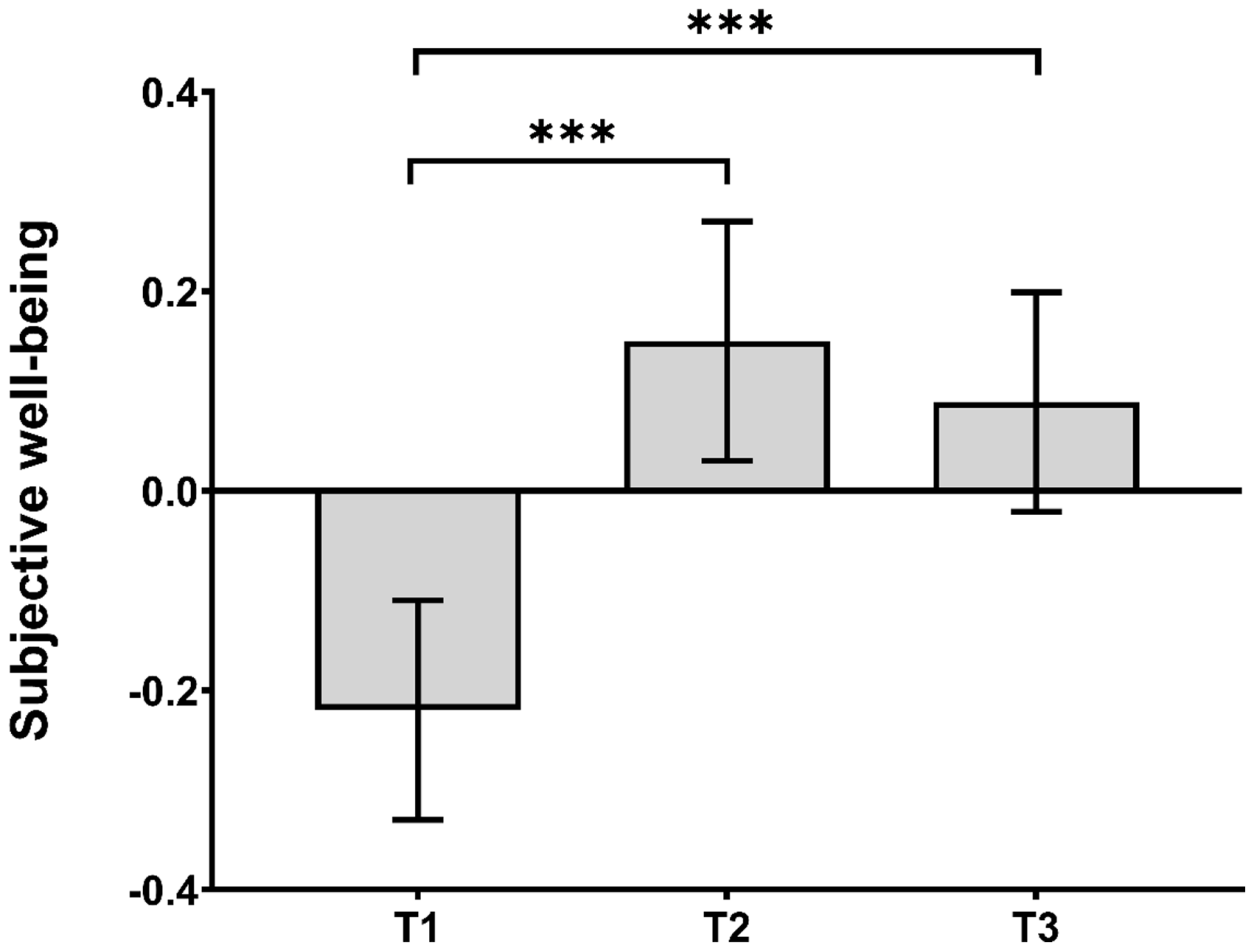
Subjective well-being of students at the T1, T2, and T3. The error bars represent standard errors. ^***^p < 0.001.

We conducted a one-way ANOVA using time as the within-subject factor (T1, T2, T3) to examine the effect of national music lessons on university students’ subjective well-being. As the outcome of Mauchly’s test of sphericity was significant (*p* < 0.001), statistics should be reported with Greenhouse–Geisser correction. There was a significant effect of time, *F* (1.91, 688.71) = 11.03, *p* < 0.001, partial eta^2^ = 0.03. Multiple comparisons (Bonferroni corrected) revealed that students’ subjective well-being at T1 (*M* = −0.22, *SE* = 0.11) was significantly lower than at T2 (*M* = 0.15, *SE* = 0.12, *p* < 0.001) and T3 (*M* = 0.08, *SE* = 0.11, *p* < 0.001). From T2 to T3, there was no change in the students’ subjective well-being (*p* = 1.000). The results are depicted in Figure 1.

### 3.2. National identity and self-esteem of university students

A one-way ANOVA was conducted to evaluate the impact of national music lessons on university students’ national identity, with time (T1, T2, T3) as the within-subject factor. The result of Mauchly’s test of sphericity was not significant (*p* = 0.063). There was no significant effect of time, *F* (2, 722) = 2.19, *p* = 0.112, partial eta^2^ < 0.01. It was unnecessary to carry out multiple comparisons.

Similarly, a one-way ANOVA using time as the with-subject factor (T1, T2, T3) was conducted to test the influence of national music lessons on university students’ self-esteem. The result of Mauchly’s test of sphericity revealed significance (*p* = 0.003), and consequently we report statistics with Greenhouse–Geisser correction. There was no significant effect of time, *F* (1.94, 700.15) = 2.20, *p* = 0.111, partial eta^2^ < 0.01. It was no need to conduct multiple comparisons.

### 3.3. Exploratory analyses on university students’ subjective well-being

The above results have demonstrated that national music lessons have an impact on subjective well-being, but no influence on self-esteem or national identity. To further explore whether there are individual differences in the influence of national music lessons on subjective well-being, we carried out a series of exploratory analyses. The participants were distinguished depending on their ethnic identity, self-esteem, and subjective well-being at T1, which were identified as three levels: high, medium, and low. As per accepted psychometric standards, those in the upper 27% are classified as high level, those in the lower 27% are classified as low level, and those in between are regarded as middle level.

We conducted a 3 (group: low national identity, middle national identity, high national identity) × 3 (time: T1, T2, T3) mixed-measures ANOVA to determine whether the various levels of national identity had an impact on national music lessons influencing subjective well-being. Mauchly’s test of sphericity was significant (*p* < 0.001), and we present statistics with Greenhouse–Geisser correction. The main effect of time was significant, *F* (1.91, 686.21) = 9.74, *p* < 0.001, partial eta^2^ = 0.03. Similarly, the main effect of group was also significant, *F* (2, 359) = 29.93, *p* < 0.001, partial eta^2^ = 0.14. Nevertheless, the interaction of time and group was not significant, *F* (3.82, 1372.42) = 1.08, *p* = 0.365, partial eta^2^ < 0.01. There was not necessary to conduct a simple effect analysis. Multiple comparisons (Bonferroni corrected) showed that students with high national identity had higher subjective well-being (*M* = 0.91, *SE* = 0.18) than those with middle national identity (*M* = 0.01, *SE* = 0.14, *p* < 0.001) and low national identity (*M* = −1.18, *SE* = 0.20, *p* < 0.001). Students with middle national identity had higher subjective well-being than those with low national identity (*p* < 0.001).

To explore whether self-esteem had an impact on national lessons affecting subjective well-being, a 3 (group: low self-esteem, middle self-esteem, high self-esteem) × 3 (time: T1, T2, T3) mixed-measures ANOVA was conducted. Mauchly’s test of sphericity was significant (*p* < 0.001), and statistics should be reported with Greenhouse–Geisser correction. Results indicated a significant main effect of time, *F* (1.91, 684.46) = 9.74, *p* < 0.001, partial eta^2^ = 0.03, as well as a significant main effect of group, *F* (2, 359) = 111.09, *p* < 0.001, partial eta^2^ = 0.38. However, the interaction of time and group was not significant, *F* (3.81, 1368.92) = 0.582, *p* = 0.668, partial eta^2^ < 0.01. It was not necessary to carry out a simple effect analysis. Multiple comparisons (Bonferroni corrected) showed that students with high self-esteem had higher subjective well-being (*M* = 1.71, *SE* = 0.16) compared to those with middle self-esteem (*M* = −0.14, *SE* = 0.12, *p* < 0.001) and those with low self-esteem (*M* = −1.63, *SE* = 0.16, *p* < 0.001). Students with middle self-esteem had higher subjective well-being than those with low self-esteem (*p* < 0.001).

**Figure 2:**
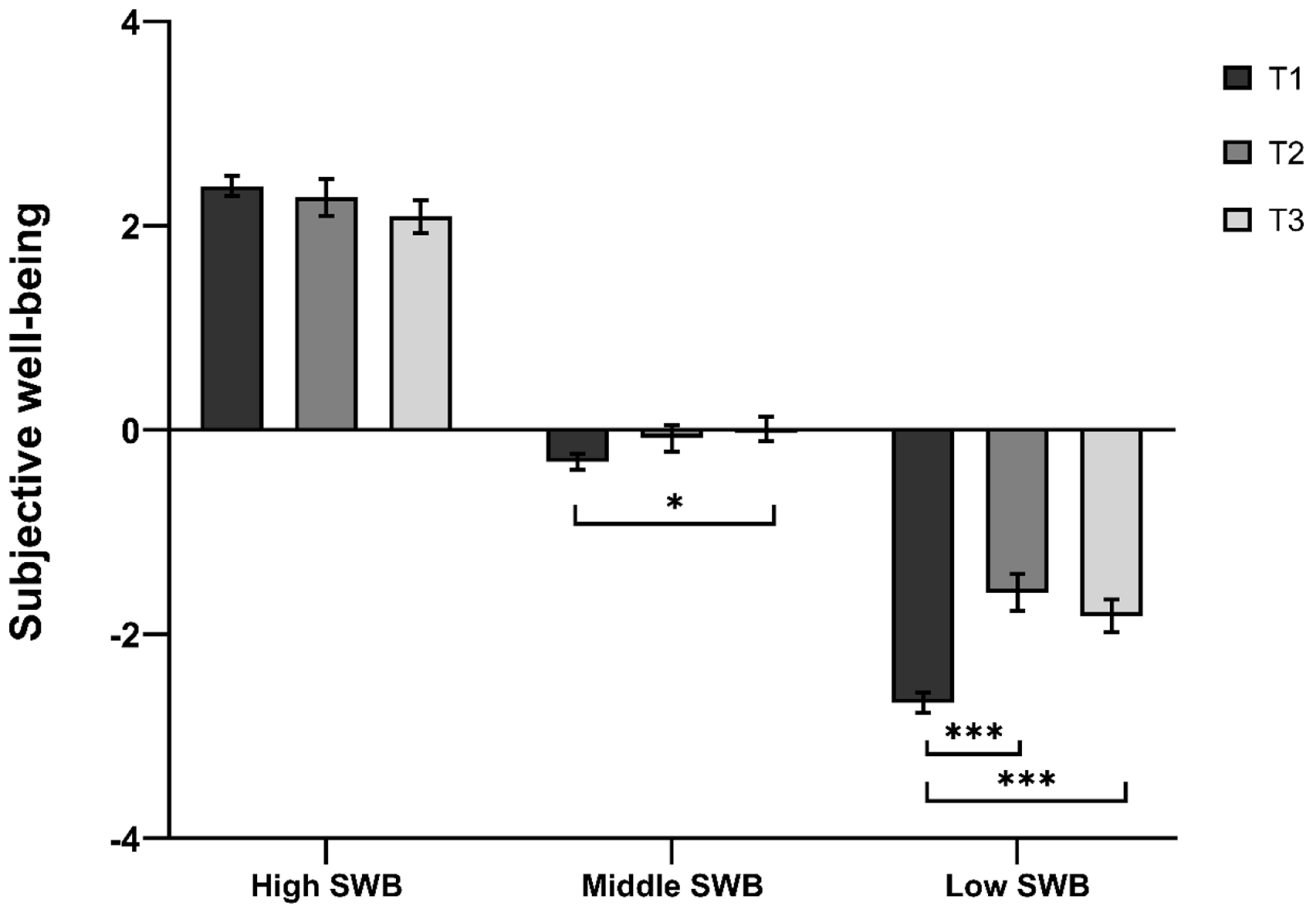
Subjective well-being for students with different levels of subjective well-being at the T1, T2, and T3. High SWB represents subjective well-being for students with high subjective well-being. Middle SWB represents subjective well-being for students with middle subjective well-being. Low SWB represents subjective well-being for students with low subjective well-being. The error bars represent standard errors. ^*^p < 0.05, ^***^p < 0.001.

To examine if the effects of national music lessons on subjective well-being were suitable for students with different levels of subjective well-being, a 3 (group: low subjective well-being, middle subjective well-being, high subjective well-being) × 3 (time: T1, T2, T3) mixed-measures ANOVA was conducted. Mauchly’s test of sphericity demonstrated significance (*p* < 0.001), and we report statistics with Greenhouse–Geisser correction. Results revealed a significant main effect of time, *F* (1.91, 685.97) = 12.29, *p* < 0.001, partial eta^2^ = 0.03, a significant main effect of group, *F* (2, 359) = 346.23, *p* < 0.001, partial eta^2^ = 0.66, and a significant interaction between the two, *F* (3.82, 1371.94) = 10.72, *p* < 0.001, partial eta^2^ = 0.06. A simple effects analysis (Bonferroni corrected) showed that students with high subjective well-being had no significant difference across T1 (*M* = 2.39, *SE* = 0.10), T2 (*M* = 2.28, *SE* = 0.18), and T3 (*M* = 2.09, *SE* = 0.16, all *p*s > 0.09). For students with low subjective well-being, national music lessons can enhance their subjective well-being from T1 (*M* = −2.69, *SE* = 0.10) to T2 (*M* = −1.59, *SE* = 0.18, *p* < 0.001) and T3 (*M* = −1.82, *SE* = 0.16, *p* < 0.001). There was no significant difference from T2 to T3 for students with low subjective well-being (*p* = 0.440). For students with middle subjective well-being, national music lessons can enhance their subjective well-being from T1 (*M* = −0.31, *SE* = 0.08) to T3 (*M* = 0.01, *SE* = 0.12, *p* = 0.010). There was no significant difference from T1 to T2 (*M* = −0.08, *SE* = 0.13, *p* = 0.249), and T2 to T3 (*p* = 1.000) for students with low subjective well-being (Figure 2).

## 4. Discussion

Based on a case from China, the current longitudinal study aims to explore the influence of national music lessons on university students’ national identity, self-esteem, and subjective well-being. The results reveal that national music lessons can enhance students’ subjective well-being, but there was no impact on their self-esteem or national identity. In addition, our exploratory analyses demonstrate that national music lessons can only improve subjective well-being for students with low or middle levels of subjective well-being, not for those with high levels. Self-esteem and national identity do not impact the influence of national music lessons on subjective well-being.

### 4.1. National music lessons enhance the subjective well-being of university students

This study verifies H1 that national music lessons can enhance the subjective well-being of university students. Previous research has established the efficacy of music listening as a strategy to regulate emotions (Randall et al., 2014). During music lessons, teachers provide their students with opportunities to listen to and enjoy music, which helps students to cope with their stress and anxiety. Furthermore, music can facilitate the implementation of other emotion regulation strategies such as distraction and reappraisal (Carvalho et al., 2022). Through national music lessons, students can divert their attention away from negative experiences or unpleasant feelings they have experienced in their daily lives. Music listening in class has a relaxing effect on the students, making them feel at ease (Saarikallio et al., 2017). Through national music lessons, students can experience improvements in their emotional well-being and life satisfaction.

The results of this study indicate that national identity and self-esteem have correlations with personal subjective well-being. Those with a strong sense of national identity or a high level of self-esteem tend to experience greater subjective well-being. The findings are consistent with previous research. Higher national identity is related to stronger emotional connection, thus contributing to greater subjective well-being (Canto and Vallejo-Martín, 2021). Self-esteem has a spillover effect on subjective well-being, with an increase in self-esteem causing an enhancement of one’s subjective well-being (Pierce et al., 2015). However, national identity and self-esteem do not appear to affect the influence of national music lessons on university students’ subjective well-being. It has been observed that regardless of the level of self-esteem and national identity, national music lessons have a similar effect on the students’ subjective well-being. Music lessons have a varied impact on students depending on their subjective well-being. Specifically, students with low or middle subjective well-being can benefit from taking music lessons. Students with high subjective well-being remain unaffected by music lessons. It appears that there is an upper threshold for happiness experience, making it hard to enhance the pleasure of those who already possess a high degree of subjective well-being.

### 4.2. National music lessons have no effect on self-esteem and national identity

This study demonstrates that national music lessons cannot affect the self-esteem and self-identity of university students. H2 and H3 should be refused. The formation of national identity in adolescence is a lasting trait (Newman, 2005). Thus, this sense of belonging and responsibility towards the national group remains stable in adults. Despite the national music course providing students with an understanding of national music and culture, it is unlikely to cause a significant change in a stable trait for adults within 8 weeks. Self-esteem is relatively stable for adults, but not immutable for long periods (Orth and Robins, 2014). The self-esteem of university students through the short-term national music course is hard to improve because of other stable determinants factors like socioeconomic status, physical attractiveness, and the degree of intimacy (von Soest et al., 2016).

### 4.3. Implications, limitations, and suggestions for future research

This study has outstanding practical value, in addition to its theoretical implications. In theory, this study further expands the relationship between music and subjective well-being, proving that national music lessons can promote university students’ subjective well-being. Additionally, it has been observed that those with a strong sense of national identity and high self-esteem tend to experience greater subjective well-being. Practically, this paper encourages universities to set up national music courses and other music courses, which are beneficial to the mental health of college students. Music teachers should take the initiative to instruct students on how to listen to and appreciate music, as it may lead to physical and mental relaxation.

This research has certain limitations. First, the sample of this study only involves university students. It is uncertain whether national music lessons have any impact on the self-esteem, national identity, and subjective well-being of adolescents. Second, this case study is conducted at a university in China. It can be concluded that Chinese national music has a beneficial impact on the mental health of Chinese university students. However, whether this conclusion is applicable to other countries should be further verified in practice. Third, this research is solely dedicated to exploring the effect of national music lessons on national identity, self-esteem, and subjective well-being, without taking into account other psychological characteristics and indicators. It is the opinion of music educators in many countries that taking music lessons is highly beneficial for students’ mental health (Law and Ho, 2011; Behr et al., 2016; Lu, 2022), yet this assertion must be confirmed through practical outcomes.

In the future, a cross-cultural study could be conducted to examine the influence of national music lessons on students, exploring the similarities and differences between various cultures or countries. Incorporating the methods of developmental and educational psychology, a long-term tracking design can be implemented to assess the impact of music lessons on various student stages. Furthermore, future studies can examine the influence of music lessons on mental health, like reducing depression/anxiety and strengthening psychological resilience. Music can effectively promote cognitive function (Schneider et al., 2019), which can also be further explored in future studies within the sphere of music education.

## Data availability statement

The raw data supporting the conclusions of this article will be made available by the authors, without undue reservation.

## Ethics statement

The studies involving human participants were reviewed and approved by Guangzhou University Institutional Review Board. The patients/participants provided their written informed consent to participate in this study.

## Author contributions

HF and JT: led writing, conceptualization, methodology, and provided reviews. HF: oversaw data analyses and led data analyses. All authors contributed to the article and approved the submitted version.

## Funding

This study was supported by the MOE (Ministry of Education in China) Youth Project of Humanities and Social Sciences (Grant No. 2022YJC760092), the Guangzhou Philosophy and Social Sciences “14th Five-year” Plan in 2021 “Yangcheng Youth Scholars” Project (Grant No. 2021GZQN14), the Guangzhou Philosophy and Social Sciences General Project (Grant No. GD22CYS04), and the Special Funds for the Cultivation of Guangdong College Students’ Scientific and Technological Innovation (Grant No. pdjh2023b0420).

## Conflict of interest

The authors declare that the research was conducted in the absence of any commercial or financial relationships that could be construed as a potential conflict of interest.

## Publisher’s note

All claims expressed in this article are solely those of the authors and do not necessarily represent those of their affiliated organizations, or those of the publisher, the editors and the reviewers. Any product that may be evaluated in this article, or claim that may be made by its manufacturer, is not guaranteed or endorsed by the publisher.
